# Identification and validation of basement membrane-related genes predicting prognosis and immune infiltration associated with bladder cancer

**DOI:** 10.1097/MD.0000000000038858

**Published:** 2024-07-19

**Authors:** Fie Lai, Lin He, Thongher Lia, Zhen Yang, Chaoyou Huang

**Affiliations:** a Department of Urology Surgery, Chengdu Second People’s Hospital, Chengdu, Sichuan, China; b Department of Pathology, Chengdu Second People’s Hospital, Chengdu, Sichuan, China.

**Keywords:** basement membrane, bladder cancer, BM-related genes, cancer, prognostic biomarker

## Abstract

Bladder cancer (BC) is fatal during muscle invasion and treatment progress is limited. In this study, we aimed to construct and validate basement membrane (BM)-associated gene prognosis to predict BC progression and tumor immune infiltration correlation. We choreographed BM-related genes in the Cancer Genome Atlas (TCGA) database using COX regression and least absolute shrinkage and selection operator (LASSO) analysis, and the predictive value of BM-related genes was further validated by the GSE32548, GSE129845, and immunohistochemistry staining. All analyses were performed with R-version 4.2.2, and its appropriate packages. Three genes were identified to construct a gene signature to predictive of BC prognosis. We divided the TCGA database into 2 groups, and patients in the high-risk group had worse overall survival (OS) than those in the low-risk group. In GSE32548, we confirmed that patients in the high-risk group had a poorer prognosis compared to those in the low-risk group in terms of OS. Immunohistochemical staining of EPEMP1, GPC2, and ITGA3 showed significantly higher expression at the protein level in BC tissues than in normal tissues. The Spearman analysis showed risk score was positively correlated with B cell naïve, Macrophages M2, and Mast cells resting. stromal score, immune score, and ESTIMATE scores were significantly higher in the high-risk group. drugs sensitivity analysis showed IC50 of Cisplatin, Gemcitabine, and Methotrexate in the high-risk group was significantly higher than that in the low-risk group. We identified 3 prognostic genes from a novel perspective of BM genes as effective risk stratification tools for BC patients.

## 1. Introduction

Bladder cancer (BC) is the most common malignant tumor of the urinary system, with the incidence ranking first among the malignant tumors of the urinary system.^[1,2]^ About 3-quarters of bladder malignancies are non-muscle-invasive.^[3]^ In contrast, 20% to 25% of bladder malignancies are muscle-invasive at the initial onset.^[4,5]^ Patients with localized disease can be cured by surgical resection or radiotherapy, but this treatment is limited if the disease is recurrent or spread over long distances.^[6]^ Despite advances in chemotherapy regimens such as platinum and 5-FU therapy for patients with advanced BC, the efficacy of chemotherapy remains unsatisfactory, the overall survival (OS) rate at 5 years was barely >50%.^[7,8]^ Targeted therapy is therefore the future of targeted BC. Targeted agents have been developed in recent years, but overall results remain disappointing. There is a need to develop valuable biomarkers to predict the possibility of reducing late-stage disease.

The basement membrane (BM) is a characteristic structural formation of the extracellular matrix and is found in a variety of tissues.^[9]^ BM is highly bioactive because it is rich in components that promote cell adhesion, growth, migration, invasion, and differentiation through a variety of cell surface receptors.^[10]^ A recent study identified up to 200 genes described to act differently at different locations in the BM.^[11]^ These genes are involved in tumorigenesis development and metastasis.^[12]^ Therefore, the measurement of these BM-related genes provides a new method for the prediction of the long-term prognosis of cancer patients.

In this study, we explored the potential clinical utility of BM-related genes prognostic stratification and targeted BC immunotherapy. We analyzed BM-related gene expression and corresponding clinical information to develop personalized prognostic models for BC patients. Bioinformatics analysis of BM-related genes was performed to explore its potential regulatory mechanisms. Our findings provide a basis for developing personalized therapies for BC patients.

## 2. Materials and methods

### 2.1. BC data source

We obtained RNA sequencing data (RNA-seq FPKM), somatic mutation data, copy number variation data, and associated clinicopathological information for breast cancer from the UCSC Xena website (UCSC Xena at xenabrowser.net). To validate the Cancer Genome Atlas (TCGA) dataset results, we additionally procured expression profiling data from both breast cancer patients and normal tissues through “GSE32548”^[13]^ and 10X Genomics single-cell RNA sequencing data from “GSE129845”^[14]^ via the gene expression omnibus (GEO) (https://www.ncbi.nlm.nih.gov/geo/) (see Fig. S1, supplementary material, Article Flowchart). http://links.lww.com/MD/N195

### 2.2. Identification of differentially expressed genes (DEGs)

We download 222 BM genes from (https://bmbase.manchester.ac.uk/). To identify DEGs in normal and tumor BM genes, we used criteria for were identified using the “limma” package in R with inclusion criteria of adjusted *P* value < .01 and |log Fold-Change| ≥ 1 for differential genes.

### 2.3. Functional enrichment analysis of DEGs

Based on these DEGs, gene ontology (GO) and Kyoto Encyclopedia of Genes and Genomes (KEGG) analysis were performed using the “clusterProfiler,”“enrichment plot,” and “ggplot 2” software package. The screening criteria adjusted *P* value < .05 for GO and KEGG enrichment analysis were statistically significant for enrichment. using GSEA (version 4.3.2) analysis to assess the KEGG enrichment pathway in patients in high and low-risk groups.

### 2.4. Construction of the BM-related gene prognostic and risk model

Univariate cox-regression analysis for DEGs was used to screen for prognosis-related genes with prognostic significance at a *P* value < .01. The screened prognosis BM-related genes were then included in the least absolute shrinkage and selection operator (LASSO) regression model, which was performed using the R package “glmnet”， in which all prognosis BM-related genes were penalized to prevent the overfitting effects of the model. The penalty parameter (λ) of the model was determined by 10-fold cross-validation that followed the minimum criterion. Thereafter, genes with independent prognosis were screened by multivariate cox-regression analysis and risk scores were calculated. The risk signature was constructed by multiplying the linear combination of the BM-related gene expression levels following algorithm: (expression level of gene1 × corresponding coefficient1 + expression level of gene2 × corresponding coefficient2+... expression level of gene n × corresponding coefficient n). Based on the results of the calculated risk score, patients were divided into high-risk and low-risk groups according to the median risk score. Validation of candidate genes at the protein level was performed through the HPA database (https://www.proteinatlas.org/)

### 2.5. Evaluation of a nomogram

For the purpose of comparing OS between the high- and low-risk groups, the TCGA database constructed the Kaplan–Meier (KM) curve with a log-rank test using the R package “survminer.” The receiver operating characteristic (ROC) curve analysis was utilized to evaluate the prediction accuracy of the BM gene via the R package “timeROC.” The validations were performed simultaneously in the GEO database. a nomogram was constructed using the R packages “rms” and “regplot” and the independent prognostic factors found in the TCGA database by means of univariate and multivariate cox-regression analysis. The availability of this nomogram was evaluated by the concordance index and calibration curve. The ROC analysis was also used to determine the nomogram accuracy in OS prediction.

### 2.6. Immunohistochemistry (IHC)

For IHC, At Chengdu Second People Hospital, tissues from 12 BC patients were obtained along with tissues from paracarcinomas. Following dewaxing and hydration, tissue slices were put in a PBS buffer. After soaking the regions in hydrogen peroxide at ambient temperature, the primary antibody was left to incubate for an entire night at 4°C. After that, the slices were tethered with hematoxylin and treated with a secondary antibody. Subsequently, the slices were sealed with neutral gum after being dehydrated using ethanol at varying concentration gradients. Next, we used a microscope to study the tissue morphology and take pictures of it. The details for the 4 antibodies used in this study are provided in Table S1(see Table S1, supplementary material, the 3 immunohistochemical antibodies). http://links.lww.com/MD/N195 The indexes of IHC include the expression intensity and the percentage of positive cells. The expression intensity is divided into 4 grades: 0 = negative, 1 = weak positive, 2 = moderate positive, and 3 = strong positive, and the percentage of positive cells refers to the proportion of positive cells in the same type of cells. The whole section was viewed under low magnification (4×) to observe the coloration of tumor parenchyma cells and the distribution of positive, select the areas of high expression, and count the percentage of positive cells among the same type of cells in 5 high magnification (20 × or 40×) fields as the result of the whole section, and the results of the interpretation were reported in percentage.

### 2.7. Single-cell data download and organization

R software and the correlation package were used to evaluate BC single-cell sequencing data. In GSE129845, single-cell sequencing data of BC were read and Seurat objects were generated. Several low-quality cells were eliminated, including those with gene expression counts < 500, mitochondrial gene expression >5%, erythrocyte gene expression >5%, and removal of the cell cycle influence on the outcomes. The data was normalized using the “LogNormalize” method. 2000 extremely distinct genes were chosen using the “FindVariableFeatures” approach. The 2000 extremely distinct genes’ principal components analysis (PCA) was downscaled using the “RunPCA” tool. The uniform manifold approximation and projection (UMAP) dimensionality reduction approach was used to show the clustered cells on a 2-dimensional map.

### 2.8. Immune infiltration and drug sensitivity analysis

The abundance of the 22 immune cells was calculated by the CIBERSORT algorithm with 1000 permutations. *P* < .05 there were statistically significant. We assessed the variations between the high- and low-risk groups in the expression levels of immune-related markers and the quantity of 22 immune cells. Immune scores and stromal scores of patients with BC were calculated by the R package “Estimation” using the ESTIMATE algorithm. relationship between assessment and risk score prognostic characteristics using immune checkpoints (ICPs) and immune suppressive cytokines (ISC). To investigate the high and low-risk groups in the clinical efficacy of chemotherapeutic agents in patients, Using the “pRRophetic” package, we were able to determine the half-inhibitory concentration (IC50) values of widely used drugs. A statistically significant *P* value was defined as <.05.

## 3. Result

### 3.1. Differentially expressed BM-related genes

**Figure 1.**
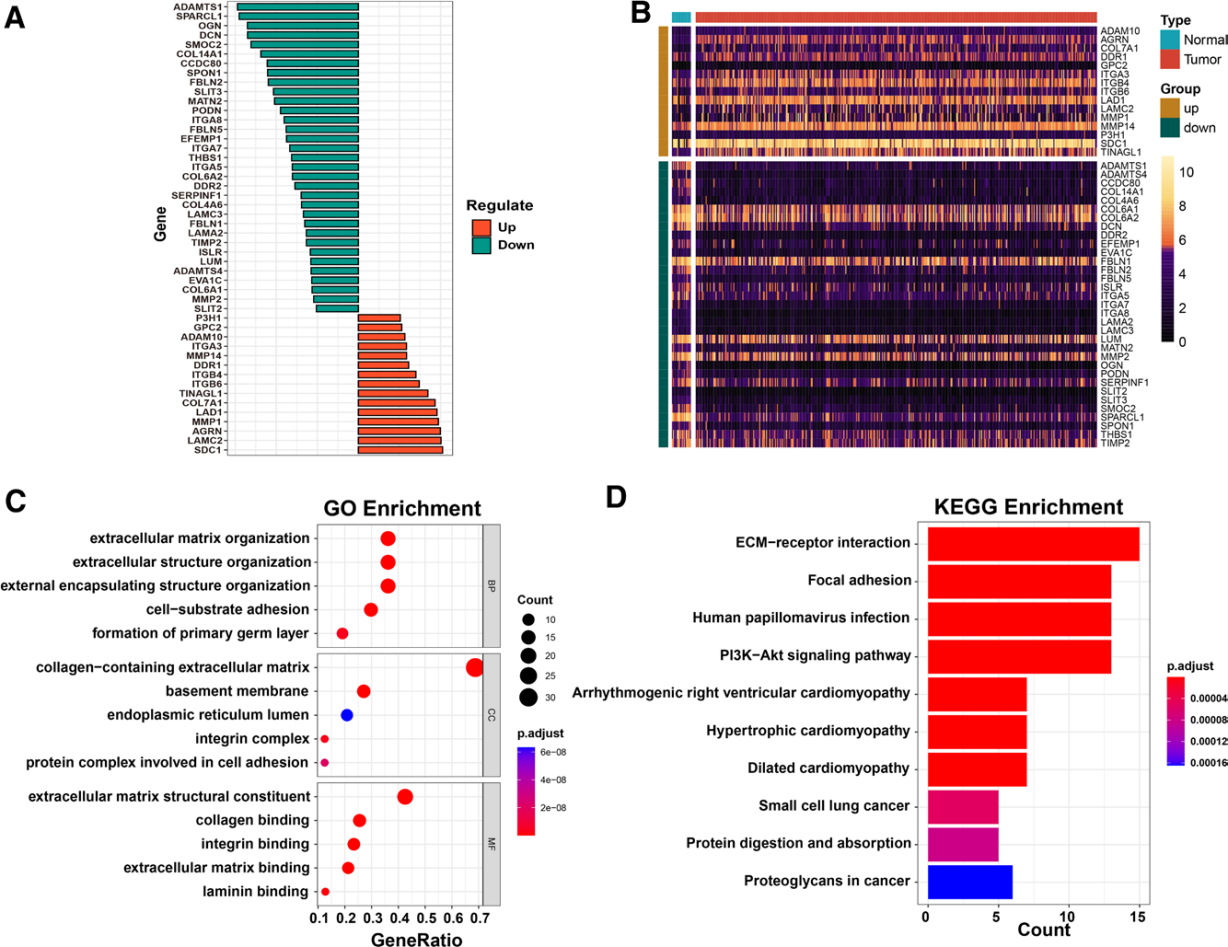
Identification and functional enrichment enrichment analysis the differentially expressed BM-related genes. (A) Histogram of differentially genetically up- and down-regulated genes in BM-related genes. (B) Heatmap of the differentially expressed BM-related genes. (C) Top 5 of GO functional enrichment analysis. (D) Top KEGG pathway analysis. BM = basement membrane, GO = gene ontology, KEGG = Kyoto Encyclopedia of Genes and Genomes.

We compared the differential expression of 222 BM-related genes in BC in 19 normal samples and 409 tumor samples (see Table S2, supplementary material, which shows the BM-related genes) http://links.lww.com/MD/N195, and we obtained a total of 48 differential genes, of which 15 were upregulated and 33 were down-regulated (Fig. 1A–B).

### 3.2. GO and KEGG pathway enrichment

Based on these deg for GO enrichment analysis and KEGG pathway analysis, the result is that there are 1150 genes enriched in GO (Table S3). http://links.lww.com/MD/N195 The results of the GO enrichment analysis of the top 5 genes showed that in the biological processes, cellular components, and molecular function (Fig. 1C). The results of KEGG pathway enrichment analysis showed that 20 enrichment pathways were mainly involved and (Table S4). http://links.lww.com/MD/N195 The top 10 ranked major pathways: ECM-receptor interaction, focal adhesion, human papillomavirus infection, PI3K-Akt signaling pathway, arrhythmogenic right ventricular cardiomyopathy, hypertrophic cardiomyopathy, dilated cardiomyopathy, small cell lung cancer, protein digestion and absorption, and proteoglycans in cancer (Fig. 1D).

### 3.3. Construction of BM-related genes prognostic model

**Figure 2.**
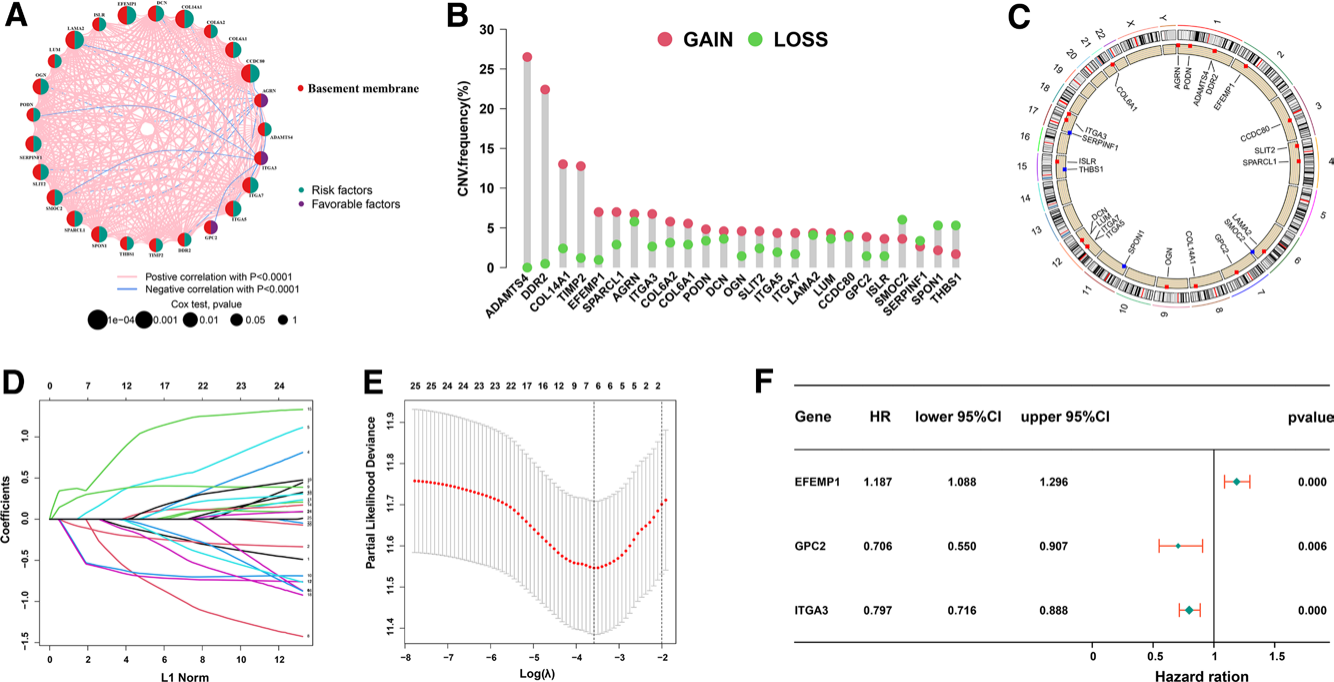
Genes associated with BM and prognosis. (A) Univariate analysis result of a network correlations in the TCGA cohort (B) Frequencies of CNV gain, loss in BM-related gene. (C) Circus plots of chromosome distributions of BM-related gene. (D, E) LASSO regression analysis of the genes from univariate analysis. (F) Results of the multivariate analysis. BM = basement membrane, CNV = copy number variation, LASSO = Least absolute shrinkage and selection operator, TCGA = The Cancer Genome Atlas.

**Figure 3.**
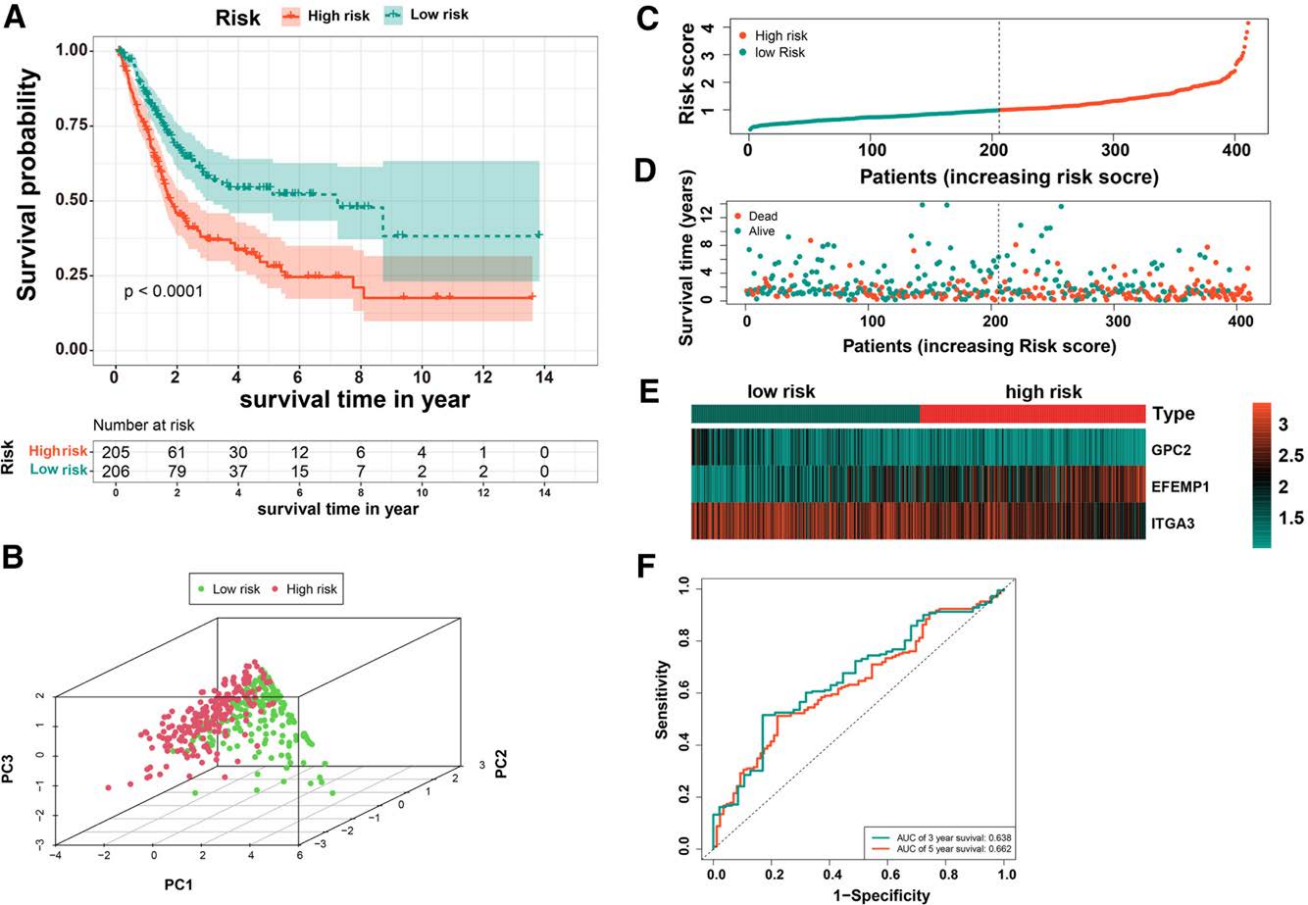
Construction of the risk score. (A) Survival probability based on the high and low-risk group. (B) PCA analysis in patients with high and low-risk groups of TCGA database. (C, D) Distribution of the survival, risk score, and survival of patients with BC. (E) Heatmap of the expression of 3 BM-related genes in BC. (F) The ROC curve for the 3- and 5-yr overall survival. BC = bladder cancer, BM = basement membrane, PCA = principal components analysis, ROC = receiver operating characteristic, TCGA = The Cancer Genome Atlas.

Univariate cox-regression analysis revealed a significant association between 25 DEGs and BC prognosis (Fig. 2A–C, see Table S5, supplementary material, http://links.lww.com/MD/N195 which shows univariate analysis). only 6 DEGs (AGRN, COL6A1, EFEMP1, GPC2, ITGA3, LAMA2) were identified as predictors after LASSO regression analysis (Fig. 2D and E). In addition, multivariate cox-regression analysis identified only 3 DEGs (EFEMP1[hazard ratio {HR}: 1.187, 95%CI: 1.088–1.296, *P* < .01], GPC2 [HR: 0.706, 95%CI: 0.550–0.907, *P* < .01], and ITGA3 [HR: 0.797, 95%CI: 0.716–0.888, *P* < .01]) used to construct prognostic models of BC patients (Fig. 2F, see Fig. S2, supplementary material, shows the KM survival analysis of 3 patient genes) http://links.lww.com/MD/N195. Following a median risk score, patients were categorized into high- and low-risk groups and subjected to a KM survival analysis (Table 1). The risk score was calculated as: Risk score = (0.171585498921286) * EFEMP1 + (−0.347995962870904) * GPC2 + (−0.226752721489659) * ITGA3. The KM survival curves showed poor prognostics for high-risk patients compared to low-risk patients (Fig. 3A). PCA, the results showed that patients in both high and low-risk groups had a significant dispersion (Fig. 3B). The risk of death increased with the increase in risk score, but patients’ survival time decreases continuously (Fig. 3C–E). The area under the curve (AUC) values for the ROC curve predicted 3-year and 5-year survival were 0.638 and 0.662, respectively (Fig. 3F).

**Table 1:** The correlations between risk groups and clinical parameters in the TCGA database.

Characteristic	Risk groups		*P* value
	High	Low	
Age			<.001
≤60	10 (13.5%)	38 (38.8%)	
>60	64 (86.5%)	60 (61.2%)	
Gender			.317
Female	20 (27.0%)	19 (19.4%)	
Male	54 (73.0%)	79 (80.6%)	
T_stage			.12
T1	0 (0%)	1 (1.0%)	
T2	17 (23.0%)	38 (38.8%)	
T3	46 (62.2%)	47 (48.0%)	
T4	11 (14.9%)	12 (12.2%)	
N_stage			<.001
N0	36 (48.6%)	82 (83.7%)	
N1	14 (18.9%)	8 (8.2%)	
N2	23 (31.1%)	7 (7.1%)	
N3	1 (1.4%)	1 (1.0%)	
M_stage			.246
M0	69 (93.2%)	96 (98.0%)	
M1	5 (6.8%)	2 (2.0%)	
Fustat			<.001
Alive	24 (32.4%)	72 (73.5%)	
Death	50 (67.6%)	26 (26.5%)	

TCGA = The Cancer Genome Atlas.

### 3.4. Prediction model

**Figure 4.**
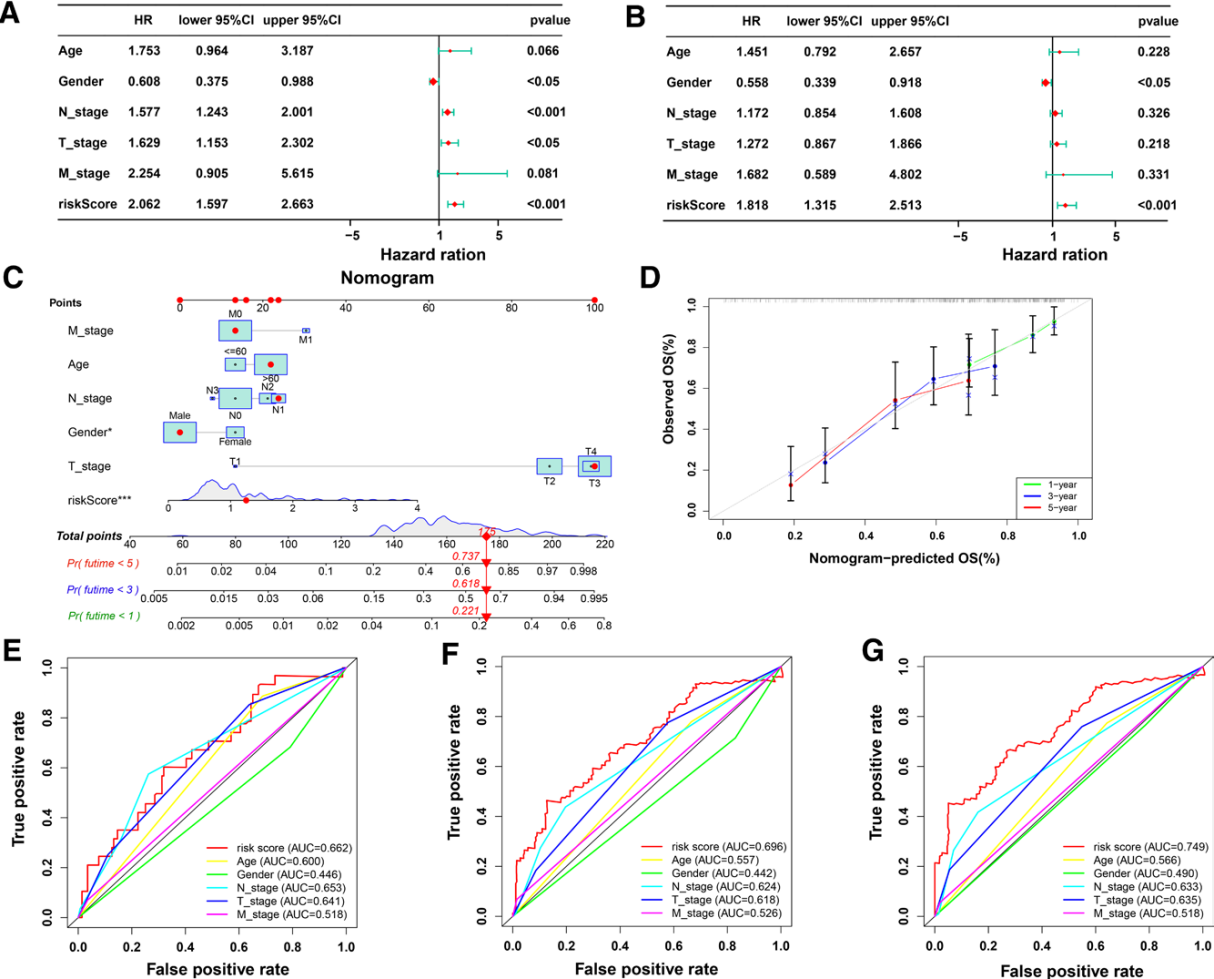
Model predictions. (A) Univariate analysis of the clinicopathologic features and the risk score. (B) Multivariate analysis of the clinicopathologic features and the risk score. (C) Nomogram to predict the survival of the BC patients. (D) Calibration curve for 1-, 3-, and 5-yr survival. (E-G) The ROC curves for multiple clinical factors and risk scores compared for 1-, 3-, and 5-yr OS in BC. BC = bladder cancer, OS = overall survival, ROC = receiver operating characteristic

In consideration of the clinical and pathological characteristics and risk score, a prediction nomogram was created. The risk score (HR = 2.062, 95%CI:1.597–2.663, *P* < .01), T-stage (HR = 1.629, 95%CI:1.153–2.302, *P* < .01), N-stage (HR = 1.577, 95%CI:1.243–2.001, *P* < .01) and gender (HR = 0.608, 95%CI:0.375–0.988, *P* = .044) were strongly correlated with prognosis, according to the univariate analysis (Fig. 4A). The risk score (HR = 1.818, 95%CI:1.315–2.513, *P* < .01), and gender (HR = 0.558, 95%CI:0.339–0.918, *P* < .05) were independent indicators that could accurately predict individuals with BC who would have a poor prognosis, according to multivariate analysis (Fig. 4B). The age, gender, N-stage, T-stage, M-stage, and risk score p values for the Schoenfeld individual test were, in order, 0.4444, 0.9848, 0.6198, 0.7672, 0.4514, and 0.7793. The global Schoenfeld test generated a *P* value of .6823 (see Table S6, supplementary material, http://links.lww.com/MD/N195 shows the global Schoenfeld test, see Fig. S3 supplementary material, http://links.lww.com/MD/N195 shows the Schoenfeld individual test). The results indicated that all of the factors were satisfied for the PH assumption. Compared to the other clinicopathological parameters, the predictive nomogram showed a higher ability to predict 3- and 5-year OS (Concordance = 0.697, Fig. 4C). The nomogram can reliably predict survival, according to the calibration curve for 1-, 3-, and 5-year survival (Fig. 4D). In addition, we did ROC curves for multiple clinical factors age, TNM staging, and risk score and calculated the area under the ROC curve (AUC) for 1-,3-, and 5 years. We found that the AUC values of risk score and other clinical factors were compared and found significantly higher AUC values for the mentioned risk score, which implies that risk score is a good prognostic predictor (Fig. 4E–G).

### 3.5. Validation of risk score and immunohistochemical analysis

**Figure 5.**
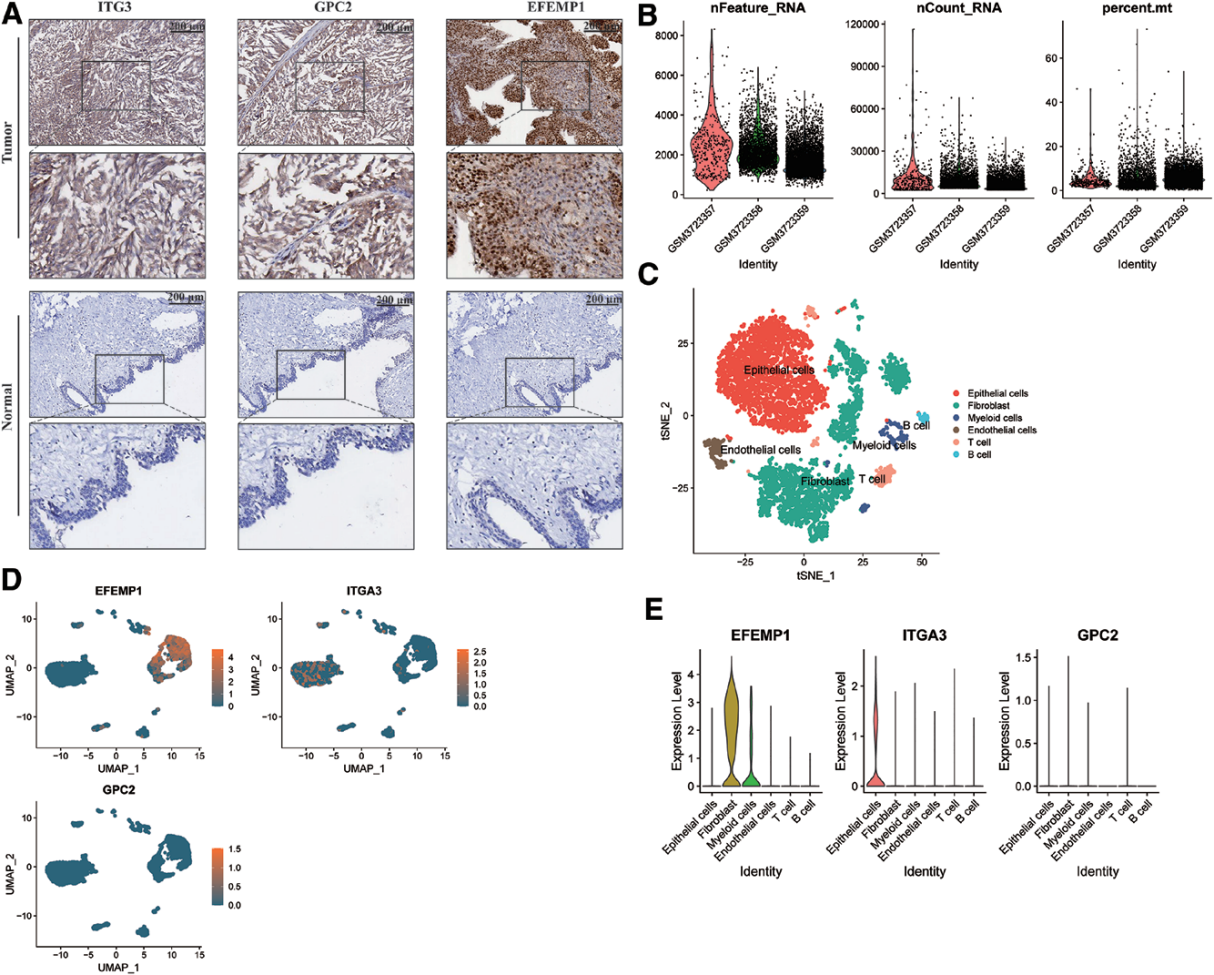
The 3 genes of immunohistochemical staining expression and expression of single-cell RNA types. (A) Expression of 3 genes in normal and tumor sample in the BC. (B) Gene expression in 3 bladder cancer single-cell RNA samples. (C-E) Tthe 3 genes of expression of single-cell RNA types. BC = bladder cancer.

The validity of the model was verified by GEO validation data. The validation dataset KM survival curve revealed that high-risk groups had a noticeably worse prognosis than low-risk groups (see Fig. S4A, supplementary material, survival probability based on the high and low-risk group) http://links.lww.com/MD/N195. In the validation dataset, 3 prognostic genes showed significant expression. The GEO validation dataset showed a drop in survival time but an increase in death risk with a higher risk score, similar to the TCGA dataset (see Fig. S4B–D, supplementary material, which shows the validation of the risk score) http://links.lww.com/MD/N195. The ROC curve areas for predicting OS patient at 3 and 5 years were 0.698 and 0.709 (see Fig. S4E, supplementary material, which shows the area under the OR curve). http://links.lww.com/MD/N195 BC patients of high and low-risk groups could be completely separated by the PCA (see Fig. S4F, supplementary material, http://links.lww.com/MD/N195 which shows the PCA analysis) based on these 3 BM-related gene with differential mRNA levels. Immunohistochemical staining showed that the GPC2 and ITG3 genes were predominantly expressed in the cytoplasm and cell membrane and were more highly expressed in BC samples than in normal samples. The EFEMP1 gene was predominantly expressed in the cytoplasm and nucleus and was also more highly expressed in BC samples than in normal samples (Fig. 5A, see Table S7, supplementary material, http://links.lww.com/MD/N195 which shows immunohistochemical staining results).

### 3.6. Single-cell RNA-Seq data analysis

To move forward investigate the identification of 3 BM-related genes in BC cells and determine their expression in specific cell types, we analyzed single-cell data (Fig. 5B). The findings showed that single-cell analysis was unable to detect the expression of the GPC2 gene, and that ITG3 was mostly expressed in epithelial cells and fibroblasts. (Fig. 5C–E).

### 3.7. Relationship between BM-related gene signature and immune cell infiltration

**Figure 6.**
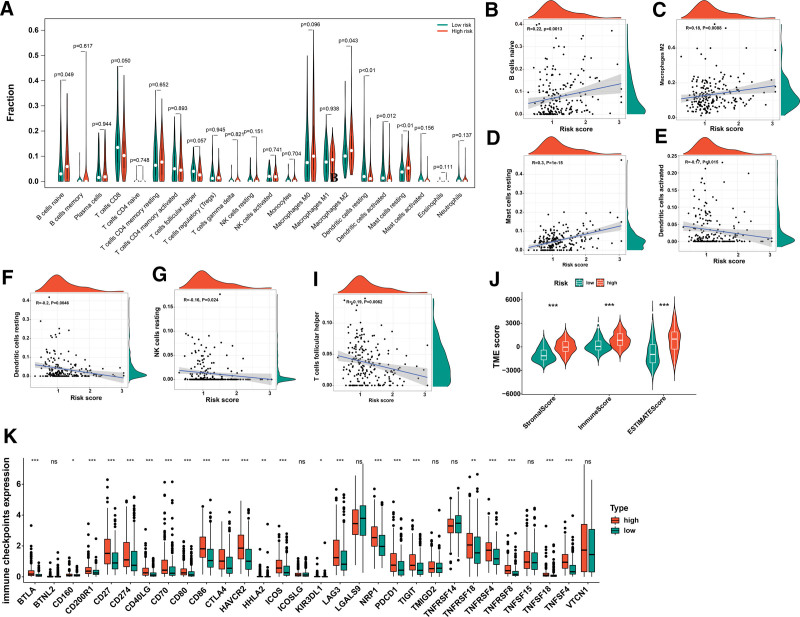
Evaluation of the TME and checkpoints. (A) Relation of the immune cells with a risk score. (B-I) Correlations between risk score and immune cell types. (J) Correlations between risk score and both immune and stromal scores. (K) Correlations between the ICPs in the high and low-risk groups (*P* < .05 *; *P* < .01 **; *P* < .001 ***). ICPs = immune checkpoints, TME = tumor microenvironment.

Using the CIBERSORT algorithm estimation, we assessed the infiltration of the 22 different types of immune cells in the TCGA data and found B cells naïve, Macrophages M2, Dendritic cells resting, Dendritic cells activated, Mast cells resting were significantly different between the high- and low-risk groups (Fig. 6A). the correlation between risk score and immune cell abundance. As depicted in Figure 6B to I, the risk score was positively correlated with B cell naïve, Macrophages M2, Mast cells resting, while risk score showed the opposite relationship with follicular Dendritic cells activated, Dendritic cells resting, NK cells resting and T cells follicular helper. The ESTIMATE score analysis result showed stromal score, immune score, and ESTIMATE scores were significantly higher in the high-risk group (Fig. 6J). Next, the relationship between ICPs and this prognostic feature was evaluated. Figure 6K shows that 30 ICPs behave differently in 2 risk subgroups, PD-1, PDL-1, and CTLA4. In addition, the relationship between ISCs and high and low-risk groups was evaluated and IL10, IL13, TGFB2 and TGFB3 were found to be statistically different in the 2 groups (see Fig. S5, supplementary material, http://links.lww.com/MD/N195 which shows expression of ISCs in the high and low-risk groups).

### 3.8. Cancer-related gene mutation and drug sensitivity analysis

**Figure 7.**
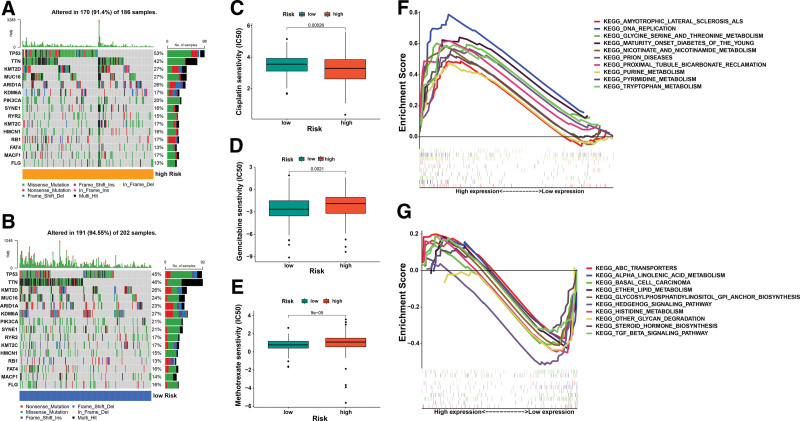
The gene mutation, drug sensitivity and GSEA analysis. (A, B) The waterfall plot of top 15 gene somatic mutation features established with high and low-risk groups. (C-E) Relationships between risk score and chemotherapeutic sensitivity. (F) The top 10 pathways of GSEA enrichment analysis in the high-risk groups. (G) The top 10 pathways of GSEA enrichment analysis in the low-risk groups.

To evaluate differences in associated with cancer mutations comparing high-risk and low-risk groups, we first estimated the mutation frequency of each group genes. Figure 7A to B describe in general the representative gene mutations found in all of the groups. Genes such as TP53 (53%), TNN (42%), KMT2D (27%), MUC16(27%), and ARID1A (26%) had the top 5 mutation frequencies in the high-risk group. TP53 (45%), TNN (46%), KMT2D (26%), MUC16(24%), and ARID1A (23%) consisted among the top 5 genes in the low-risk category with the greatest frequency of mutations. The TCGA dataset estimation of the chemotherapy response in the 2 distinct groups of risks. We identified BC most often used medications, such as Cisplatin, Gemcitabine, Camptothecin, Doxorubicin, Thapsigargan, and Methotrexate. The results show that the IC50 of Cisplatin, Gemcitabine, and Methotrexate was considerably greater within the high-risk group from the TCGA dataset than that observed in the low-risk group (Fig. 6C–E), This indicates that individuals in both categories can respond differently to these chemotherapeutic drugs. Likewise, the variations in IC50 for camptothecin, Doxorubicin, Thapsigargan, and Vinblastine in both groups were not significant (see Fig. S6, supplementary material, shows the risk score and chemotherapeutic sensitivity) http://links.lww.com/MD/N195.

### 3.9. GSEA enrichment analysis

To investigate any variations in signaling pathways comparing the 2 patient groups that have different risk. GSEA was performed. Our results demonstrated that the majority of high-risk patients enriched in metabolic signaling pathways (Fig. 7F), such as glycine serine and threonine metabolism, nicotinate and nicotinamide metabolism, purine metabolism, pyrimidine metabolism, and tryptophan metabolism. Moreover, the low-risk group was enriched with ABC transporters, alpha-linolenic acid metabolism, basal cell carcinoma, ether lipid metabolism, glycosylphosphatidylinositol GPI anchor biosynthesis, hedgehog signaling pathway, histidine metabolism, other glycan degradation, steroid hormone biosynthesis, TGF beta signaling pathway (Fig. 7G).

## 4. Discussion

BC is one of the most lethal tumors in man.^[15,16]^ Although diagnostic and treatment techniques have advanced, the overall incidence of BC has been progressively increasing.^[17]^ it remains a disease on which human beings need concentrate. BM cleavage is critical for tumor invasion and progression, clearing the way for tumor breakthroughs and crossing mechanical barriers in the first place.^[18]^ The early phases of malignant epithelial transition in the BC are marked by deformation of normal structures and loss of cell polarity, which is connected with tumor aggressiveness.^[19,20]^ This is caused by changes to the BM protein half-chromosomes and receptors, which are found in the membranes of malignant cells. Altered integrins lose their role as determinants and maintainers of tissue integrity and facilitate further progression. These receptors are reassigned to membrane protrusions in order to promote adhesion, migration, and survival in invading tissues.^[21]^

In the present investigation, we determined the transcriptional alterations and expression of BM-based genes in the TCGA-BLCA cohort. Our findings revealed that prognostic models based on EFEMP1, GPC2 and ITGA3 could reliably forecast BC patients’ prognosis, and constructed correlation models and risk scores for prognosticating breast cancer, which will aid in the comprehension of the disease molecular pathophysiology and improve comprehension of diagnosis, therapy, and prognosis. The fact that individuals with a high BM score also had an unfavorable OS is noteworthy, as it implies that a high BM score could be a predictor of an unfavorable outcome. BM is connected to the harmful actions of several tumors.

The epidermal growth factor-containing fibulin-like extracellular matrix protein 1 (EFEMP1) high expression correlated with adverse pathologic characteristics of UC and independently predicted adverse outcomes.^[22]^ EFEMP1 promotes angiogenesis and promotes cervical cancer growth through VEGF upregulation.^[23]^ GPC2 is a prognostic marker for numerous cancers and an adjuvant to early tumor diagnosis.^[24]^ Researchers found that GPC2 expression was upregulated in prostate cancer and small cell lung cancer suggesting poor patient prognosis.^[25,26]^ Furthermore, experience suggests that high expression of GPC2 in duct adenocarcinoma after pancreaticoduodenectomy may lead to favorable prognosis.^[27]^ One study found that by stimulating the miR-1184/ITGA3 axis, circBC048201 encourages the growth, migration, and invasion of BC cells.^[28]^ In breast cancer, ITGA3 is a novel biomarker to evaluate breast cancer diagnosis and prognosis. In addition, ECM modulation and immune cell infiltration are mediated by ITGA3.^[29]^ MiR-223 downregulation improved ITGA3/ITGB1 signaling and aided in PCa cells’ ability to migrate and invade cancer cells.^[30]^ Our immunohistochemical staining showed that these 3 genes were expressed higher in bladder tumor samples than in normal samples. The findings of the studies we mentioned above support our results.

The application of risk scores to predict the prognosis of prognostic tumors has been shown,^[31,32]^ and In BC patients, our risk score was substantially correlated with survival. The findings imply that among BC patients, the risk score is a reliable indicator of survival outcomes. Its 3- and 5-year OS prediction robustness was proven using ROC. Furthermore. univariate and multifactorial cox-regression analyses of BC pathological clinical characteristics and risk scores were significantly correlated. The multifactor ROC curve area was predictive of 1-, 3-, and 5-year. Consequently, risk ratings might be a good indicator of a patient prognosis. Finally, the risk score prognosis was validated using the GEO dataset, and the outcomes demonstrated that the risk score could predict prognosis with accuracy. We, therefore, think that this could be a novel, highly accurate predictive model.

Immune cell infiltration is associated with BC occurrence, prognosis, and treatment. For a number of years, immunotherapy has considerably extended the period that patients with advanced BC survive,^[33]^ The immune system and stromal cells are essential elements of the tumor microenvironment, and immune and stromal scores associated with clinical characteristics and prognosis in BC.^[34,35]^ The stromal and immunological scores of the high-risk group were greater than those of the low-risk group, according to our calculations using the ESTIMATE program execution. This suggested that BM could be associated with tumor microenvironment involvement to regulate neoplastic invasion and migration, disease progression. Treatment for clear cell renal carcinoma includes ICP inhibition, and progress in this area is still happening quickly.^[36,37]^ ICP analysis showed that most high-risk groups were significantly higher than low-risk groups, which may suggest a correlation between BM and ICP inhibitor therapy, such as for advanced bladder urothelial carcinoma, either with or without active PI3K pathway, the combination of ICB with Pan-PI3K inhibition exerts strong antitumor effects.^[38]^ study shows that muscle-invasive urothelial carcinoma treated with ICP inhibitor nivolumab is more beneficial in disease-free survival patients compared to controls.^[39]^ Cisplatin, Gemcitabine, and Methotrexate which are chemotherapeutic agents for oncology, are standard treatments for patients with advanced tumors and can improve survival and progression in BC patients.

The PI3K-Akt signaling pathway and ECM-receptor interaction are linked to the results of the functional enrichment analysis of the BMs-related genes using KEGG. Receptor interaction has been shown to have a critical role in the development of cancer and the production of metastases.^[40,41]^ Studies have also been reported in BC the ECM-receptor interaction pathway plays a role in the development of BC and may be involved in the progression of BC.^[42]^ An essential signaling pathway in tumors is the PI3K-Akt pathway, and a variety of tumor development is associated with this pathway,^[43–45]^ and has been demonstrated to play a part in the onset of BC in BC.^[46,47]^

This study has several limitations. First, we did not perform in vivo and in vitro experiments to validate the results obtained from our study. Second, we did not validate BC specimens and additional in vivo and in vitro studies will be needed to confirm our findings. Despite these limitations, we believe that our results will be useful and informative for future studies on BM-related genes in BC.

## 5. Conclusions

It is possible that EPEMP1, GPC2, and ITGA3 are predictive biomarkers that are useful and that have a significant role in immune cell infiltration in people with BC. Further clinical research on this biomarker model is necessary to confirm its clinical relevance.

## Acknowledgments

We acknowledge TCGA and GEO database for providing its platforms.

## Author contributions

**Conceptualization:** Lin He, Zhen Yang.

**Data curation:** Fei Lai, Zhen Yang, Chaoyou Huang.

**Formal analysis:** Thongher Lia.

**Investigation:** Lin He.

**Methodology:** Lin He, Thongher Lia, Chaoyou Huang.

**Project administration:** Fei Lai, Thongher Lia, Chaoyou Huang.

**Resources:** Chaoyou Huang.

**Software:** Lin He, Zhen Yang.

**Validation:** Lin He, Zhen Yang.

**Writing – original draft:** Fei Lai, Thongher Lia, Zhen Yang, Chaoyou Huang.

**Writing – review & editing:** Fei Lai.

## Supplementary Material


