# Supplementary material for: Identification and validation of basement membrane-related genes predicting prognosis and immune infiltration associated with bladder cancer
Source: Medicine (Baltimore). 2024 Jul 19;103(29):e38858. doi: 10.1097/MD.0000000000038858 (PMC11398827; doi:10.1097/MD.0000000000038858)
Supplement: Supplementary file 1 [file medi-103-e38858-s001.pdf]

Table S1: Immunohistochemical (IHC) antibodies.

No.	Antibody name	Description	Reactivity	Company	Product No.	Dilute
1	Anti-GCP2 antibody	Rabbit polyclonal	Human GCP2	Affinity Biosciences	DF13374	1:100
2	Anti-ITGA3 antibody	Rabbit polyclonal	Human ITGA3	Affinity Biosciences	AF5182	1:100
3	Anti-EFEMP1 antibody	Rabbit Monoclonal	Human EFEMP1	Chengdu Zen-bioscience	R26707	1:80

Table S2: 222 Basement membrane genes

id	Category
ACAN	Basement membrane component
ACHE	Basement membrane component
ADAMTS1	Basement membrane component
ADAMTS2	Basement membrane component
ADAMTS3	Basement membrane component
ADAMTS4	Basement membrane component
ADAMTS5	Basement membrane component
ADAMTS6	Basement membrane component
ADAMTS7	Basement membrane component
ADAMTS8	Basement membrane component
ADAMTS9	Basement membrane component
ADAMTS10	Basement membrane component
ADAMTS13	Basement membrane component
ADAMTS14	Basement membrane component
ADAMTS15	Basement membrane component

ADAMTS16	Basement membrane component
ADAMTS17	Basement membrane component
ADAMTS18	Basement membrane component
ADAMTS19	Basement membrane component
ADAMTS20	Basement membrane component
AGRN	Basement membrane component
AMELX	Basement membrane component
AMTN	Basement membrane component
ANG	Basement membrane component
BCAN	Basement membrane component
BGN	Basement membrane component
CCDC80	Basement membrane component
CERT1	Basement membrane component
COL2A1	Basement membrane component
COL4A1	Basement membrane component
COL4A2	Basement membrane component

COL4A3	Basement membrane component
COL4A4	Basement membrane component
COL4A5	Basement membrane component
COL4A6	Basement membrane component
COL5A1	Basement membrane component
COL6A1	Basement membrane component
COL6A2	Basement membrane component
COL6A3	Basement membrane component
COL7A1	Basement membrane component
COL8A1	Basement membrane component
COL8A2	Basement membrane component
COL9A1	Basement membrane component
COL9A2	Basement membrane component
COL9A3	Basement membrane component
COL12A1	Basement membrane component
COL14A1	Basement membrane component

COL15A1	Basement membrane component
COL17A1	Basement membrane component
COL18A1	Basement membrane component
COL28A1	Basement membrane component
COLQ	Basement membrane component
CST3	Basement membrane component
CTSA	Basement membrane component
CTSB	Basement membrane component
CTSD	Basement membrane component
DCN	Basement membrane component
ECM1	Basement membrane component
EFEMP1	Basement membrane component
EFEMP2	Basement membrane component
EGFL6	Basement membrane component
EGFLAM	Basement membrane component
FBLN1	Basement membrane component

FBLN2	Basement membrane component
FBLN5	Basement membrane component
FBN1	Basement membrane component
FBN2	Basement membrane component
FBN3	Basement membrane component
FGF9	Basement membrane component
FMOD	Basement membrane component
FN1	Basement membrane component
FRAS1	Basement membrane component
FREM1	Basement membrane component
FREM2	Basement membrane component
FREM3	Basement membrane component
HAPLN1	Basement membrane component
HMCN1	Basement membrane component
HMCN2	Basement membrane component
HSPG2	Basement membrane component

ISLR	Basement membrane component
LAD1	Basement membrane component
LAMA1	Basement membrane component
LAMA2	Basement membrane component
LAMA3	Basement membrane component
LAMA4	Basement membrane component
LAMA5	Basement membrane component
LAMB1	Basement membrane component
LAMB2	Basement membrane component
LAMB3	Basement membrane component
LAMB4	Basement membrane component
LAMC1	Basement membrane component
LAMC2	Basement membrane component
LAMC3	Basement membrane component
LOXL1	Basement membrane component
LOXL2	Basement membrane component

LOXL4	Basement membrane component
LUM	Basement membrane component
MATN1	Basement membrane component
MATN2	Basement membrane component
MATN4	Basement membrane component
MEP1A	Basement membrane component
MEP1B	Basement membrane component
MMP1	Basement membrane component
MMP2	Basement membrane component
MMP7	Basement membrane component
MMP17	Basement membrane component
MMP21	Basement membrane component
MMP26	Basement membrane component
MMRN2	Basement membrane component
NELL1	Basement membrane component
NELL2	Basement membrane component

NID1	Basement membrane component
NID2	Basement membrane component
NPNT	Basement membrane component
NTN1	Basement membrane component
NTN4	Basement membrane component
OGN	Basement membrane component
OPTC	Basement membrane component
P3H1	Basement membrane component
P3H2	Basement membrane component
PAPLN	Basement membrane component
PODN	Basement membrane component
POSTN	Basement membrane component
PTN	Basement membrane component
PXDN	Basement membrane component
PXDNL	Basement membrane component
RECK	Basement membrane component

SERPINF1	Basement membrane component
SLIT1	Basement membrane component
SLIT2	Basement membrane component
SLIT3	Basement membrane component
SEMA3B	Basement membrane component
SMC3	Basement membrane component
SMOC1	Basement membrane component
SMOC2	Basement membrane component
SPARC	Basement membrane component
SPARCL1	Basement membrane component
SPOCK1	Basement membrane component
SPOCK2	Basement membrane component
SPOCK3	Basement membrane component
SPON1	Basement membrane component
SPON2	Basement membrane component
TGFB2	Basement membrane component

TGFB1	Basement membrane component
TGFBI	Basement membrane component
THBS1	Basement membrane component
THBS2	Basement membrane component
THBS4	Basement membrane component
TIMP1	Basement membrane component
TIMP2	Basement membrane component
TIMP3	Basement membrane component
TINAG	Basement membrane component
TINAGL1	Basement membrane component
TLL1	Basement membrane component
TNC	Basement membrane component
USH2A	Basement membrane component
VCAN	Basement membrane component
VTN	Basement membrane component
VWA1	Basement membrane component

VWA2	Basement membrane component
ADAM9	Cell surface interactor
ADAM10	Cell surface interactor
ADAM17	Cell surface interactor
CD44	Cell surface interactor
CD151	Cell surface interactor
CSPG4	Cell surface interactor
COL13A1	Cell surface interactor
DAG1	Cell surface interactor
DCC	Cell surface interactor
DDR1	Cell surface interactor
DDR2	Cell surface interactor
EVA1A	Cell surface interactor
EVA1B	Cell surface interactor
EVA1C	Cell surface interactor
GPC1	Cell surface interactor

GPC2	Cell surface interactor
GPC3	Cell surface interactor
GPC4	Cell surface interactor
GPC5	Cell surface interactor
GPC6	Cell surface interactor
ITGA1	Cell surface interactor
ITGA2	Cell surface interactor
ITGA2B	Cell surface interactor
ITGA3	Cell surface interactor
ITGA4	Cell surface interactor
ITGA5	Cell surface interactor
ITGA6	Cell surface interactor
ITGA7	Cell surface interactor
ITGA8	Cell surface interactor
ITGA9	Cell surface interactor
ITGA10	Cell surface interactor

ITGAM	Cell surface interactor
ITGAV	Cell surface interactor
ITGAX	Cell surface interactor
ITGB1	Cell surface interactor
ITGB2	Cell surface interactor
ITGB3	Cell surface interactor
ITGB4	Cell surface interactor
ITGB5	Cell surface interactor
ITGB6	Cell surface interactor
ITGB7	Cell surface interactor
ITGB8	Cell surface interactor
MEGF9	Cell surface interactor
MMP14	Cell surface interactor
MPZL2	Cell surface interactor
MUSK	Cell surface interactor
PTPRF	Cell surface interactor

RPSA	Cell surface interactor
ROBO1	Cell surface interactor
ROBO2	Cell surface interactor
ROBO3	Cell surface interactor
ROBO4	Cell surface interactor
SDC1	Cell surface interactor
SDC4	Cell surface interactor
TENM1	Cell surface interactor
TENM2	Cell surface interactor
TENM3	Cell surface interactor
TENM4	Cell surface interactor
UNC5A	Cell surface interactor
UNC5B	Cell surface interactor
UNC5C	Cell surface interactor
UNC5D	Cell surface interactor

Table S3: Top 5 enrichment for GO enrichment analysis of 48 basement membrane differential genes

ONTOLOGY	ID	Description	GeneRatio	p.adjust	Count
BP	GO:0030198	extracellular matrix organization	17/47	1.90E-16	17
BP	GO:0043062	extracellular structure organization	17/47	1.90E-16	17
BP	GO:0045229	external encapsulating structure organization	17/47	1.90E-16	17
BP	GO:0031589	cell-substrate adhesion	14/47	3.98E-11	14
BP	GO:0001704	formation of primary germ layer	9/47	3.69E-09	9
CC	GO:0062023	collagen-containing extracellular matrix	33/48	3.77E-42	33
CC	GO:0005604	basement membrane	13/48	2.75E-18	13
CC	GO:0008305	integrin complex	6/48	3.44E-09	6
CC	GO:0098636	protein complex involved in cell adhesion	6/48	2.09E-08	6
CC	GO:0005788	endoplasmic reticulum lumen	10/48	6.34E-08	10
MF	GO:0005201	extracellular matrix structural constituent	20/47	5.51E-27	20
MF	GO:0005518	collagen binding	12/47	4.65E-18	12
MF	GO:0050840	extracellular matrix binding	10/47	3.21E-15	10
MF	GO:0005178	integrin binding	11/47	3.22E-12	11
MF	GO:0043236	laminin binding	6/47	9.61E-10	6

Table S4: 20 Enrichment pathways of 48 basement membrane differential genes KEGG

ID	Description	GeneRatio	p.adjust	Count
hsa04512	ECM-receptor interaction	15/26	2.88E-22	15
hsa04510	Focal adhesion	13/26	1.52E-13	13
hsa05165	Human papillomavirus infection	13/26	6.36E-11	13
hsa04151	PI3K-Akt signaling pathway	13/26	1.12E-10	13
hsa05412	Arrhythmogenic right ventricular cardiomyopathy	7/26	2.68E-08	7
hsa05410	Hypertrophic cardiomyopathy	7/26	6.76E-08	7
hsa05414	Dilated cardiomyopathy	7/26	9.13E-08	7
hsa05222	Small cell lung cancer	5/26	5.20E-05	5
hsa04974	Protein digestion and absorption	5/26	8.04E-05	5
hsa05205	Proteoglycans in cancer	6/26	0.000165	6
hsa04810	Regulation of actin cytoskeleton	6/26	0.000212	6
hsa05146	Amoebiasis	4/26	0.001018	4
hsa05219	Bladder cancer	3/26	0.001018	3
hsa05145	Toxoplasmosis	3/26	0.017399	3
hsa04926	Relaxin signaling pathway	3/26	0.024028	3
hsa05144	Malaria	2/26	0.031909	2
hsa04912	GnRH signaling pathway	2/26	0.092929	2
hsa04350	TGF-beta signaling pathway	2/26	0.092929	2
hsa04640	Hematopoietic cell lineage	2/26	0.093624	2
hsa04933	AGE-RAGE signaling pathway in diabetic complications	2/26	0.093624	2

Table S5. Univariate analysis of the BM-related gene cohort

Gene	HR	HR.95L	HR.95H	pvalue
ADAMTS4	1.233221	1.051433	1.44644	0.009985
AGRN	0.790959	0.678022	0.922709	0.002852
CCDC80	1.259318	1.134383	1.398012	1.52E-05
COL14A1	1.279537	1.13812	1.438526	3.71E-05
COL6A1	1.179531	1.079512	1.288817	0.00026
COL6A2	1.147951	1.056962	1.246774	0.001057
DCN	1.161281	1.064887	1.266401	0.00072
DDR2	1.327313	1.120867	1.571784	0.001028
EFEMP1	1.201111	1.106231	1.304128	1.27E-05
GPC2	0.720997	0.566671	0.917352	0.007769
ISLR	1.129951	1.045662	1.221033	0.00201
ITGA3	0.862783	0.777498	0.957422	0.005447
ITGA5	1.212161	1.086752	1.352042	0.000554
ITGA7	1.242976	1.092381	1.414331	0.000964
LAMA2	1.614998	1.322308	1.972475	2.62E-06
LUM	1.117583	1.029596	1.213089	0.007882
OGN	1.259498	1.105288	1.435224	0.000536
PODN	1.199143	1.066011	1.348903	0.00249
SERPINF1	1.180219	1.07209	1.299255	0.000725
SLIT2	1.623462	1.258867	2.093652	0.000189

SMOC2	1.2030 92	1.0888 71	1.3292 96	0.0002 8
SPARCL1	1.1798 2	1.0730 07	1.2972 65	0.0006 37
SPON1	1.2099 23	1.0877 88	1.3457 7	0.0004 48
THBS1	1.1760 29	1.0630 24	1.3010 48	0.0016 57
TIMP2	1.1704 83	1.0634 86	1.2882 45	0.0012 89

Table S6: The result of the global Schoenfeld test

	**chisq**	**df**	**Pvalue**
Age	0.58485	1	0.44
Gender	0.000364	1	0.98
N_stage	1.777558	3	0.62
T_stage	1.140781	3	0.77
M_stage	0.567246	1	0.45
riskScore	0.078521	1	0.78
GLOBAL	7.45093	10	0.68

Table S7: Staining for EFEMP2, ITG3 and GPC2 protein in BC cohorts

**Expression**	**EFEMP2**		**ITG3**		**GPC2**	
	Normal n(%)	Tumor n(%)	Normal n(%)	Tumor n(%)	Normal n(%)	Tumor n(%)
0-Negative	5(41.7)	0(0)	5(41.7)	0(0)	3(25)	0(0)
1-Weak	4(33.3)	9(75)	4(33.3)	8(66.7)	4(33.3)	6(50)
2-Moderate	3(25)	2(16.7)	3(25)	3(25)	5(41.7)	5(41.7)
3-strong	0(0)	1(8.3)	0(0)	1(8.3)	0(0)	1(8.3)

Figure S1

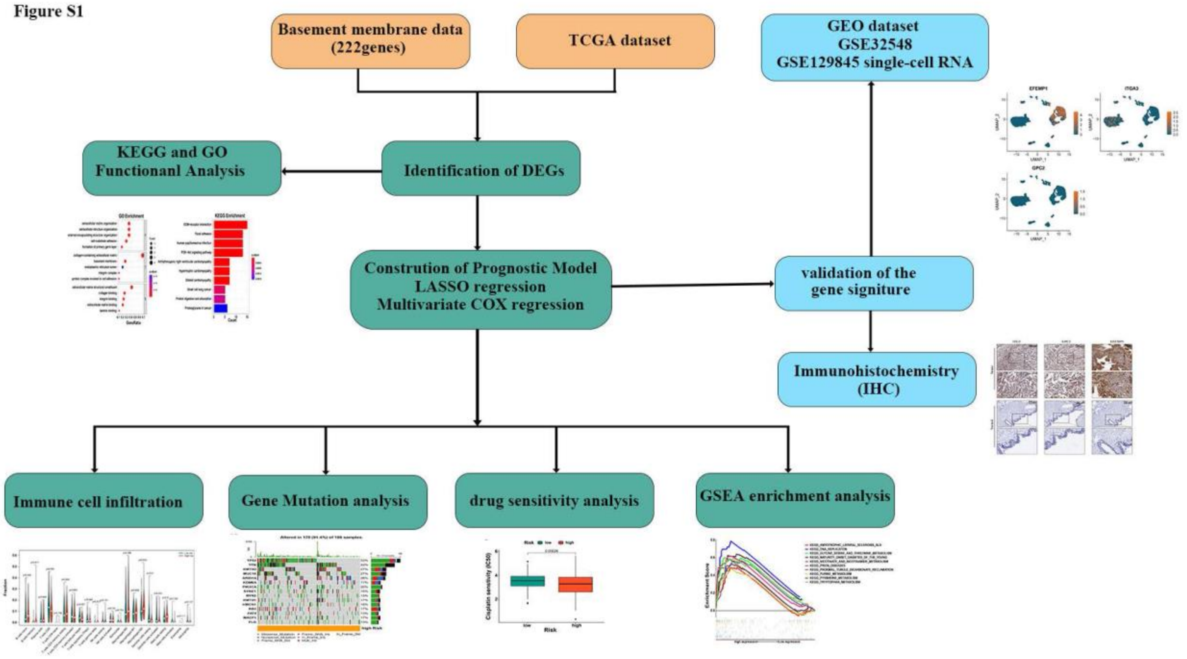
Basement membrane data
(222genes)
TCGA dataset
GEO dataset
GSE32548
GSE129845 single-cell RNA
KEGG and GO
Functionanl Analysis
Identification of DEGs
Constrution of Prognostic Model
LASSO regression
Multivariate COX regression
validation of the
gene signiture
Immunohistochemistry
(IHC)
Immune cell infiltration
Gene Mutation analysis
drug sensitivity analysis
GSEA enrichment analysis

Figure S2

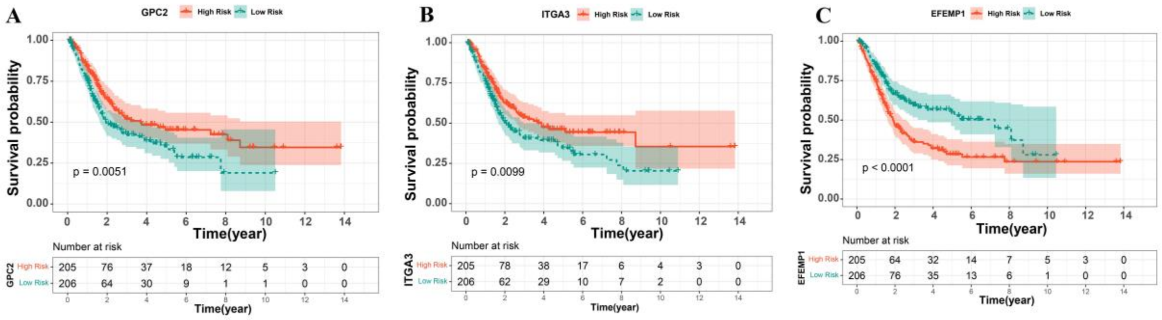
A
GPC2
High Risk
Low Risk
Survival probability
p = 0.0051
Time(year)
Number at risk
High Risk 205 76 37 18 12 5 3 0
Low Risk 206 64 30 9 1 1 0 0
B
ITGA3
High Risk
Low Risk
Survival probability
p = 0.0099
Time(year)
Number at risk
High Risk 205 78 38 17 6 4 3 0
Low Risk 206 62 29 10 7 2 0 0
C
EFEMP1
High Risk
Low Risk
Survival probability
p < 0.0001
Time(year)
Number at risk
High Risk 205 64 32 14 7 5 3 0
Low Risk 206 76 35 13 6 1 0 0

Figure S3

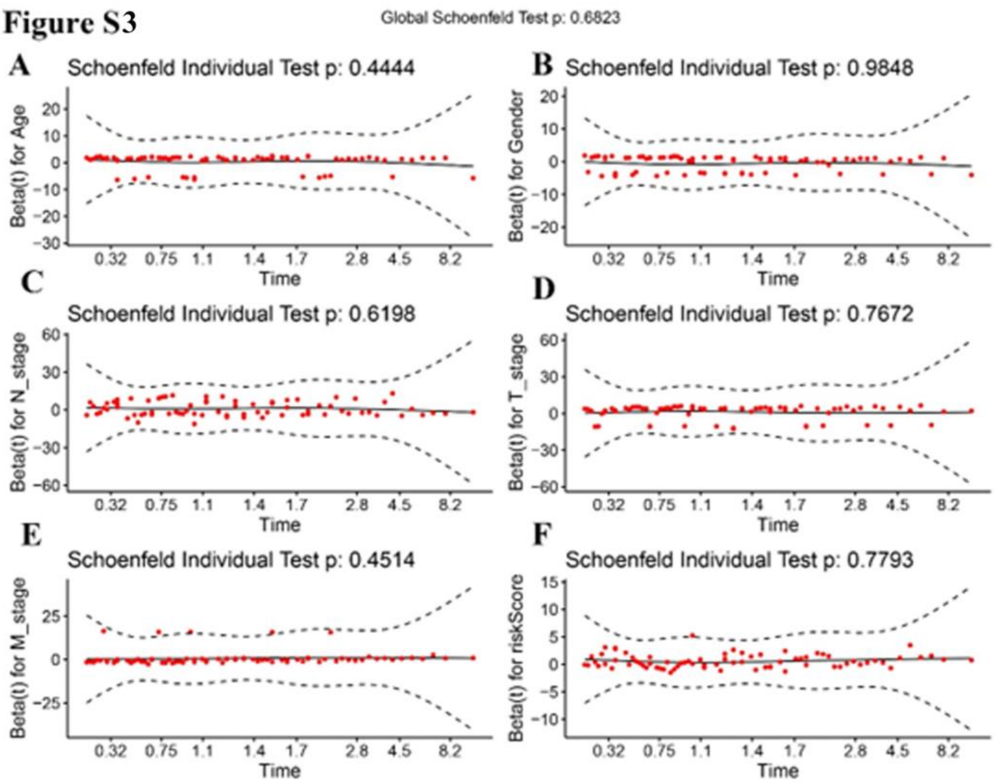
Global Schoenfeld Test p: 0.6823
A
Schoenfeld Individual Test p: 0.4444
Beta(t) for Age
Time
B
Schoenfeld Individual Test p: 0.9848
Beta(t) for Gender
Time
C
Schoenfeld Individual Test p: 0.6198
Beta(t) for N_stage
Time
D
Schoenfeld Individual Test p: 0.7672
Beta(t) for T_stage
Time
E
Schoenfeld Individual Test p: 0.4514
Beta(t) for M_stage
Time
F
Schoenfeld Individual Test p: 0.7793
Beta(t) for riskScore
Time

FigureS 4

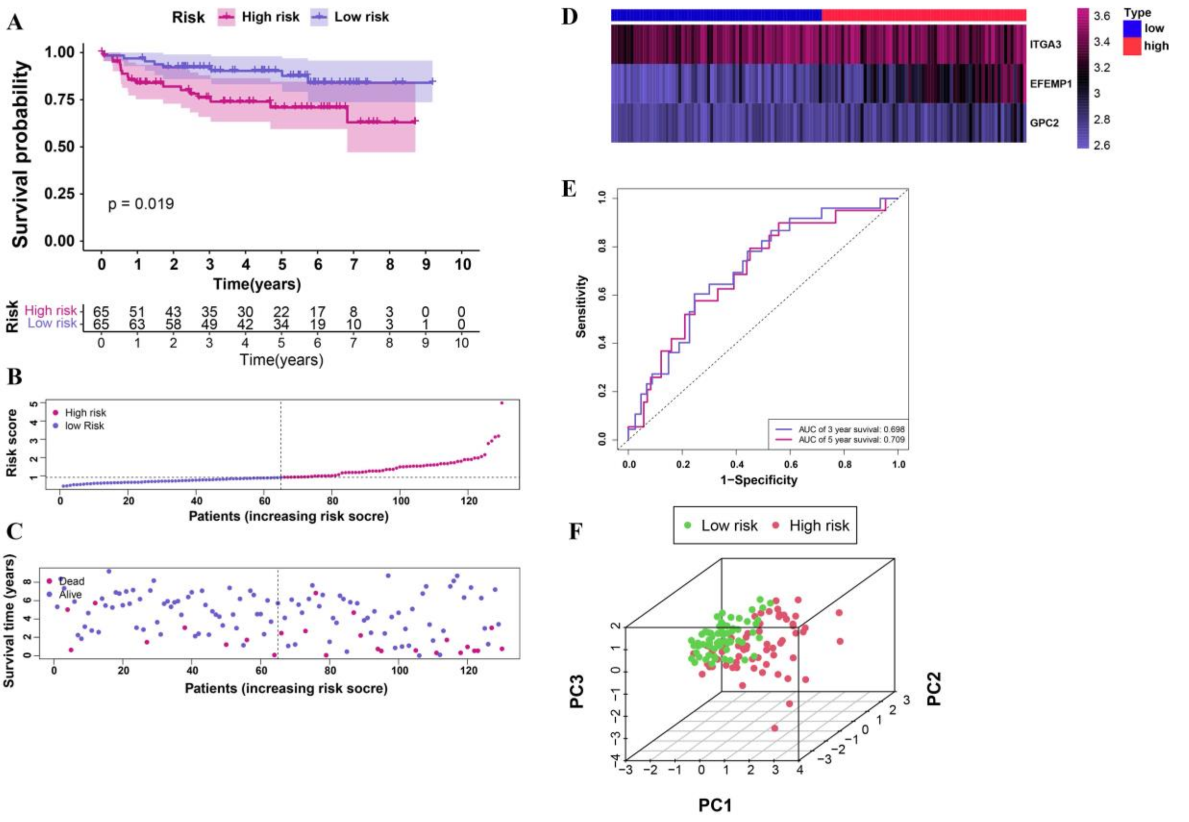

A
Risk
High risk
Low risk
Survival probability
p = 0.019
Time(years)
B
High risk
low Risk
Risk score
Patients (increasing risk socre)
C
Dead
Alive
Survival time (years)
Patients (increasing risk socre)
D
Type
low
high
ITGA3
EFEMP1
GPC2
E
Sensitivity
1−Specificity
AUC of 3 year suvival: 0.698
AUC of 5 year suvival: 0.709
F
Low risk
High risk
PC1
PC2
PC3

Figure S5

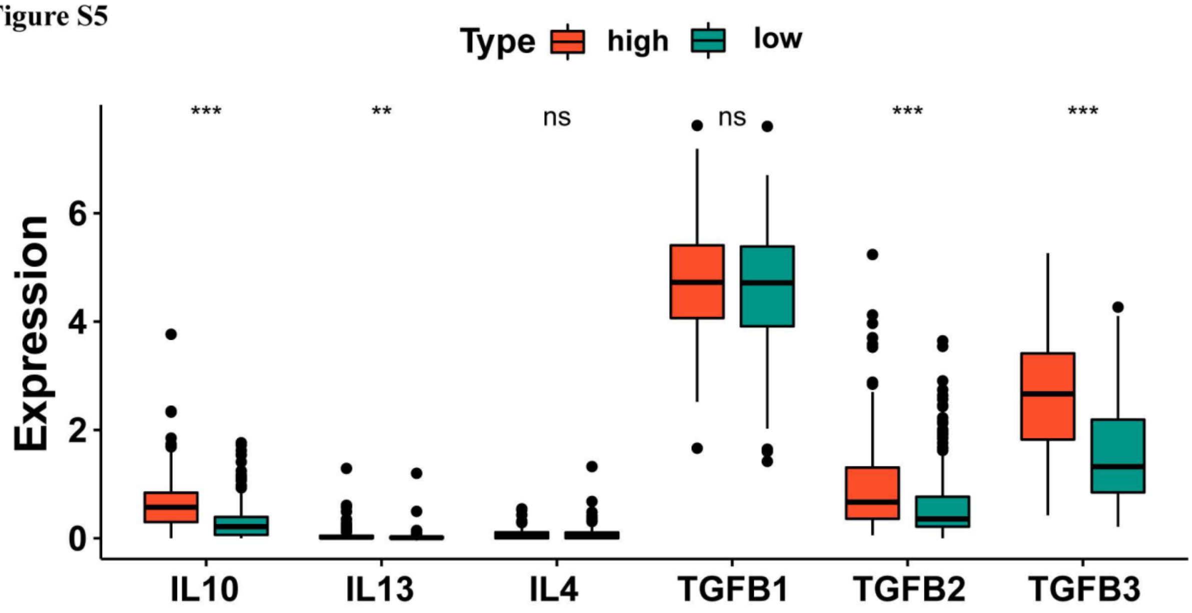
Type high low
***
**
ns
ns
***
***
Expression
0
2
4
6
IL10
IL13
IL4
TGFB1
TGFB2
TGFB3

Figure S6

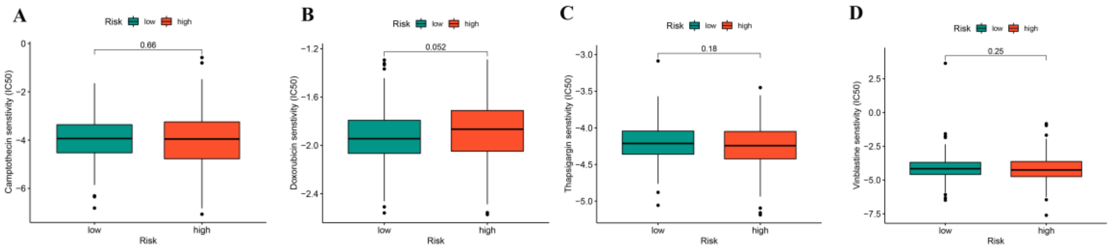
A
Risk low high
0.66
Camptothecin sensitivity (IC50)
0
-2
-4
-6
low
high
Risk
B
Risk low high
0.052
Doxorubicin sensitivity (IC50)
-1.2
-1.6
-2.0
-2.4
low
high
Risk
C
Risk low high
0.18
Thapsigargin sensitivity (IC50)
-3.0
-3.5
-4.0
-4.5
-5.0
low
high
Risk
D
Risk low high
0.25
Vinblastine sensitivity (IC50)
2.5
0.0
-2.5
-5.0
-7.5
low
high
Risk
